# First Results in the Use of Bovine Ear Notch Tag for Bovine Viral Diarrhoea Virus Detection and Genetic Analysis

**DOI:** 10.1371/journal.pone.0164451

**Published:** 2016-10-20

**Authors:** Christian Quinet, Guy Czaplicki, Elise Dion, Fabiana Dal Pozzo, Anke Kurz, Claude Saegerman

**Affiliations:** 1 Arsia, Health Department, Ciney, Belgium; 2 IFN Schönow GmbH, Bernau bei Berlin, Germany; 3 Research Unit in Epidemiology and Risk analysis applied to Veterinary Sciences (UREAR-ULg), Fundamental and Applied Research for Animal and Health (FARAH), Faculty of Veterinary Medicine, University of Liege, Liege, Belgium; Instituto Butantan, BRAZIL

## Abstract

**Background:**

Infection due to bovine viral diarrhoea virus (BVDV) is endemic in most cattle-producing countries throughout the world. The key elements of a BVDV control programme are biosecurity, elimination of persistently infected animals and surveillance. Bovine viral diarrhoea (BVD) is a notifiable disease in Belgium and an official eradication programme started from January 2015, based on testing ear notches sampled during the official identification and registration of calves at birth. An antigen-capture ELISA test based on the detection of BVDV E^rns^ protein is used. Ear notch sample may also be used to characterize the genotype of the calf when appropriate elution/dilution buffer is added. Both BVDV antigen-ELISA analysis and animal traceability could be performed.

**Methodology:**

With regards to the reference protocol used in the preparation of ear notch samples, alternative procedures were tested in terms of BVDV analytic sensitivity, diagnostic sensitivity and specificity, as well as quality and purity of animal DNA.

**Principal Findings/Significance:**

The Allflex DNA Buffer D showed promising results in BVDV diagnosis and genome analyses, opening new perspectives for the livestock industry by the exploitation of the animal genome. Due to the high number of cattle involved in the Belgian official BVDV eradication programme based on ear notch tags sample, a large database on both BVDV status of newborn calves and cattle genome could be created for subsequent different uses (e.g. traceability, determination of parentage, genetic signatures throughout the genome associated with particular traits) evolving through a more integrated animal health.

## Introduction

Bovine viral diarrhoea virus (BVDV), belonging to the *Flaviviridae* family, commonly infects cattle worldwide and causes considerable economic losses [1]. The complex epidemiology of BVDV partially lies in its ability to infect the fetus. Particularly, if the infection occurs during the period of 35–120 days of pregnancy, the virus is able to cause a persistent infection of the fetus and may result in the birth of a persistently infected (PI) calf [2] that continuously shed large amounts of BVDV in the environment. Detection and traceability of these animals are therefore a key point in any bovine viral diarrhoea (BVD) control or eradication programme [3].

In Belgium, a cross-sectional study was performed between November 2009 and March 2010. The true prevalence of BVDV-specific antibodies and antigens was respectively 47.4% and 4.4% at herd level and 32.9% and 0.3% at animal level [4].

BVD is a notifiable disease in Belgium and an official eradication programme started from January 2015 [5], based on testing ear notches sampled by farmers during the official identification and registration process of calves at birth. In several European countries, national control schemes are elaborated based on the same principle of elimination of persistently infected animals [1].

Ear notch testing is a reliable method for detecting PI animals [6] and has proven to be practical and efficient in Switzerland BVD programme [7], despite the fact that false negative ear notch test results are possible and estimated as 2.7% in Tyrol in Austria [8–9].

The tests commonly used for detecting the virus are BVDV antigen-ELISA and RT-PCR. The first one is robust, simple, cost-effective [10] and thus appropriate for testing individual samples of blood, serum, milk and ear tissue. The most two conserved and immunogenic proteins of BVDV are used as antigen, the envelope glycoprotein E^rns^ and the non-structural protein NS3 (previously named p80) [11]. Ag-ELISA targeting glycoprotein E^rns^ performed on ear notch samples is preferred to test young animals because E^rns^ Ag remains detectable also in the presence of colostral antibodies [8]. Indeed, an indirect Antigen Capture ELISA, the BVDV Ag/Serum Plus Test (IDEXX Laboratories, Inc., USA) is currently used in Belgium in the context of the mandatory official BVDV eradication program. Ear notches are sampled with the Allflex Tissue Sampling Tag system (TST) (video available on the website http://www.allflex-europe.com). The ear tissue is trapped in a needle (step 1), enclosed in a dry tube and sent to the laboratory (step 2). Once in the laboratory, the sample is extracted out of the needle and placed into a new tube (step 3), where the IDEXX buffer of the BVDV Ag/Serum Plus Test is added (step 4).

However, to determine the true PI status of an animal, two samples at an interval of minimum three weeks are necessary to differentiate PI from transient infection (TI) [12–14]. Indeed, a single positive indirect Antigen Capture ELISA can originate from a PI or a TI animal. To distinguish between PI and TI animals a second sampling is realized. The requested delay between the two samplings is at least 3 weeks as BVDV can be cleared from the blood of TI animals within 14 to 21 days post-infection [12]. Indeed, the Belgian eradication programme allows owners of BVDV positive animals to repeat the test in order to verify their permanent or transient infected status. In case of BVDV negative result obtained with the second sample, animal DNA of the two samples should be compared to confirm their genetic identity.

The ear notch tissue soaking buffer of the IDEXX BVDV Ag/Serum Plus Test is not designed to conserve DNA quality of samples. On the contrary, the Allflex DNA Buffer D (DBD) (FertiPro n.v., Beernem, Belgium) was commonly used to stabilize DNA from biopsies (data available on request to Allflex). This study was undertaken to test and validate under field conditions the use of the Allflex DNA Buffer D within the IDEXX BVDV Ag/Serum Plus Test, in order to allow BVDV diagnosis and genome analysis on the same eluted fraction. In terms of sample preparation, different protocols were tested aiming to improve laboratory logistics and evaluate the influence of the delay between sample collection and laboratory analyses.

## Materials and Methods

### Animals and samples

Ear notch tissues were sampled at birth from three groups of calves in the context of the BVDV Belgian eradication program and tested using the IDEXX BVDV Ag/Serum Plus ELISA.

The first group was constituted by 5 calves with a positive result at the antigen capture ELISA, followed by a positive PCR result at the Belgian Reference Laboratory (VAR, Uccle, Belgium), where a persistent infection with BVDV type I could be confirmed. Based on these results, the 5 calves constituted the BVDV positive PI group.

A second group of 68 calves constituted the negative control. Indeed, after initial negative results obtained on the ear notches using the antigen capture ELISA, consecutive negative test results could defined them as non-PI and not infected.

A third group was constituted by 30 calves with a BVDV positive result at birth obtained with the Ag capture ELISA. However, because the confirmation and the characterization of the positive result were not requested at the Belgian Reference Laboratory, their PI or TI condition could not be ruled out. These animals constituted a BVDV ELISA positive group.

Complete ears were sampled just after euthanasia and were directly frozen at -20°C until preparation procedure. Calves belonging to the BVDV positive PI group and the BVDV ELISA positive group were mandatory euthanized by the veterinary practitioners in the context of the Belgian BVDV eradication program. Calves of the negative control group died after birth for reasons independent from this study and were sent for necropsy to ARSIA. Notches were sampled from frozen ears and prepared on the same day (Day 0) with different protocols (see section below) and then stored at 6 ± 2°C until analysis.

### Sample preparation

According to the different objectives of the study, different protocols were applied for the ear notch preparation (**Table 1)**.

**Table 1:** Description of the protocols used in the study.

Protocol	Description
*Reference protocol*	
Protocol 1 (P1)	Allflex TST system was applied as routinely used to tag newborn calves (protocol above described). Once in the laboratory, the ear tissue was extracted from the needle into a tube where the IDEXX eluent of the BVDV Ag/Serum Plus Test (IDEXX buffer) was added.
*Alternative protocols*	
Protocol 2 (P2)	In step 4 of P1, the Allflex DNA Buffer D (Allflex buffer, produced by FertiPro N.V., Beernem, Belgium) was used instead of IDEXX buffer.
Protocol 3 (P3)	At the time of the ear tag application, the needle was closed in a tube pre-filled with IDEXX buffer.
Protocol 4 (P4)	Like P3, except for the use of Allflex DNA Buffer D instead of IDEXX buffer.
Protocol 5 (P5)	At the time of sampling, the ear notch is directly pushed in a tube pre-filled with IDEXX buffer.
Protocol 6 (P6)	Like P5, except for the use of Allflex DNA Buffer D instead of IDEXX buffer.

### BVD Antigen Diagnostic

Analytical sensitivity (the degree of response to a change in concentration of analyte being measured in an assay) was assessed by end-point dilution analysis. To evaluate the analytical sensitivity of the BVDV antigen capture ELISA, samples obtained from the BVDV positive PI group were prepared with protocols P1, P2, P3 and P4. Successive two-fold dilutions (1:2 to 1:2048) were tested 1, 3 and 14 days after the preparation (corresponding to the soaking time of the tissue in the buffer). The minimal and the median dilutions providing a positive result at the ELISA test and obtained for the five samples were calculated using the four different protocols.

To evaluate the diagnostic specificity (DSp) of the test, 68 samples originating from the negative group of calves were tested with protocols P3 and P4 at day 7 after preparation. The results were compared with those obtained with protocol P1 at day 1, which was considered as reference testing procedure. Protocols P2, P5 and P6 were not applied to the negative samples because protocols P3 and P4 allowed to assess independently the effect of both the needle and the buffer. Evaluation of the DSp occurred after 7 days of preparation in order to reflect field conditions.

To evaluate the diagnostic sensitivity (DSe) of the test, 30 samples from the BVDV ELISA positive group were prepared with protocols P3, P4, P5 and P6 and were tested at day 3, day 7 and day 28. Results were compared to those obtained with protocol P1 at day 1 as reference. The protocol P2 was not included in this analysis because the effect of Allflex buffer was evaluated via the protocol P6.

In addition, one objective was to evaluate the effect of the delay between the ear notch sampling and its successive laboratory analysis.

Independently from the protocol used in the preparation of the ear notch, all samples were tested for the detection of BVDV specific antigen, using the BVDV Ag/Serum Plus ELISA (IDEXX Laboratories, Inc., USA).

According to the manufacturer’s recommendations, samples with optical density (OD) values ≥ 0.3 were considered positive, while samples with OD values ≥ 0.2 but < 0.3 were classified uninterpretable and those with OD < 0.2 were interpreted as negative.

### Extraction of DNA and determination of its quality and quantity

In order to assess the quantity and the purity of the DNA content in the ear notch samples, a total of 18 BVDV negative and 12 BVDV positive samples were randomly selected among the negative group and the BVDV ELISA positive group, respectively.

These samples were prepared with protocols P3, P4, P5 and P6 and tested at day 28 to reproduce field conditions in Belgian eradication programme (in case of retesting). Protocols P1 and P2 were not used because the delay between the sampling of the ear notch and the addition of the buffer was considered too long to be able to protect the DNA. Indeed, to distinguish between PI and TI animals, a second sampling is realized with a minimum delay of 3 weeks. With this in mind, only day 28 was considered.

#### Extraction of DNA

Isolation of genomic DNA from the tissue samples was performed on the Kingfisher Flex platform (Thermo Fisher Scientific, Inc.) using a customised mag^TM^ DNA extraction Kit (LGC Genomics GmbH, Germany, www.lgcgroup.com/genomics). The manufacturer’s protocol for DNA extraction from hair, adapted for tissue samples was followed. Briefly, tissue was lysed for 2 h in 600 μl pre-diluted Lysis-Wash buffer C1 with 60 μl Protease K solution at 55°C in a heat incubator (MixingBlock MB-102, BIOER). Undissolved tissue was spun down at 4000 rpm in a plate centrifuge (Megafuge 16, Heraeus) and 600 μl of clear lysate was transferred into a Kingfisher binding plate containing a mixture of 300 μl Lysis-Wash buffer C1 and 60 μl fully suspension of mag particle BLM. The principle of the mag^TM^ DNA extraction Kit is a magnetic separation for the preparation of nucleic acids. Superparamagnetic particles are used to capture nucleic acids via a polarity-based binding mechanism. The following steps of the protocol, such as the washing steps to remove impurities from the sample (using 600 μl of C1, A1 or absolute ethanol) and the DNA elution from the magnetic particles (120 μl Elution buffer BLM) were performed in the automated Kingfisher platform.

#### Photometric assessment of the concentration and the purity of DNA

A Nanodrop^TM^ 1000 (Thermo Scientific) photometric was used to evaluate the quantity of extracted genomic cattle DNA in all tested samples. DNA absorption peak was measured at 260 nm, while sparse impurity induced by protein, solvent, and salt was measured between 230 nm and 280 nm.

#### Genomic DNA integrity using agarose gel electrophoresis

**Fig 1:**
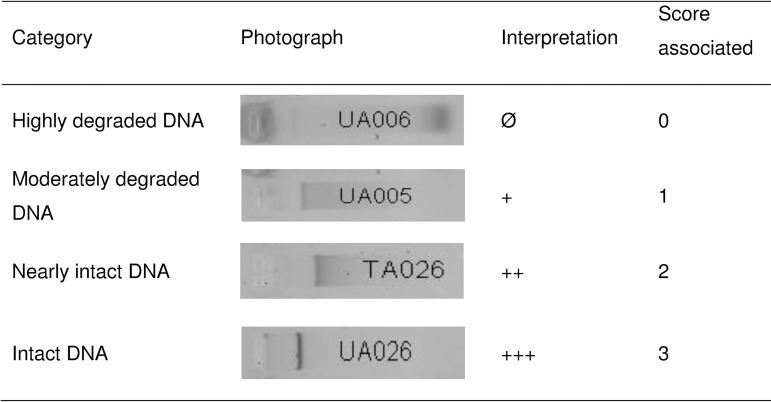
Interpretation of the genomic DNA integrity using agarose gel electrophoresis.

This technique was used to evaluate DNA degradation caused by the tested elution buffers. DNA degradation and integrity were classified in four categories: highly degraded DNA (Ø), moderately degraded DNA (+), nearly intact DNA (++) and intact DNA (+++) as shown in **Fig 1**.

### Multiplex PCR, micro-satellite typing and parentage determination of calves

An in-house accredited multiplex PCR was performed using the QIAGEN Multiplex PCR Kit (Qiagen GmbH, Germany). A mix of 5 μl of Multiplex PCR Master Mix, 1 μl of Q-Solution, 2.6 μl of RNase-Free water and 0.5 μl of a Primer-Mix containing the primers of the targeted microsatellites (2 μM of each, 4 μM for ETH3) was prepared. PCR amplification was performed on a Thermocycler T3000 by corporation Biometra using 9.1 μl of this mix and 0.9 μl of the extracted DNA, and with the following cycling programme: 15 min at 95°C, 30 cycles of 30 s at 94°C, 90 s at 58°C, 60 s at 60°C, and finally 55 min at 72°C.

Micro-satellite typing amplification was applied for parentage determination of cattle using a routine genotype panel comprising the 12 primers of the International Panel of Microsatellites for Cattle Parentage Testing (ISAG Panel) and another one recognized by NCBI. This protocol allowed to exclude the presence of inhibiting agents in the purified DNA. Amplified fragments were separated by capillary electrophoresis on an automated sequencer (ABI Prism 3130, Applied Biosystems, Life Technologies GmBH, Germany), and generated data were analyzed with Gene Mapper software v.4.0 (Applied Biosystems, Life Technologies GmBH, Germany) for allele and genotype calling. A total of 13 markers were amplified (**Table 2**) and four categories were defined based on the successful amplification of the markers: +++, when all markers were amplified; ++, when between 1 to 3 markers were not amplified; +, when between 4 to 12 markers were not amplified and Ø, when no marker (13/13) was amplified.

**Table 2:** Specific primer sequences used in the study (N = 13).

Locus	Fluorescence label		Primer Sequence (5'-3')
***ISAG Panel***			
BM1824	NED	Forward	GAG CAA GGT GTT TTT CCA ATC
	Chr 1/178-192 bp	Reverse	CAT TCT CCA ACT GCT TCC TTG
BM2113	FAM	Forward	GCT GCC TTC TAC CAA ATA CCC
	Chr 2/123-143 bp	Reverse	CTT CCT GAG AGA AGC AAC ACC
INRA023	HEX	Forward	GAG TAG AGC TAC AAG ATA AAC TTC
	Chr 3/197-223 bp	Reverse	TAA CTA CAG GGT GTT AGA TGA ACT C
SPS115	FAM	Forward	AAA GTG ACA CAA CAG CTT CTC CAG
	Chr 15/240-270 bp	Reverse	AAC GAG TGT CCT AGT TTG GCT GTG
TGLA122	HEX	Forward	CCC TCC TCC AGG TAA ATC AGC
	Chr 21/137-181 bp	Reverse (1)	AAT CAC ATG GCA AAT AAG TAC ATA C
		Reverse (2)	AAT CAC ATG GCA AAT AAG TAC ATA
TGLA126	HEX	Forward	CTA ATT TAG AAT GAG AGA GGC TTC T
	Chr 20/116-122 bp	Reverse	TTG GTC TCT ATT CTC TGA ATA TTC C
TGLA227	FAM	Forward	CGA ATT CCA AAT CTG TTA ATT TGC T
	Chr 18/76-102 bp	Reverse	ACA GAC AGA AAC TCA ATG AAA GCA
ETH10	FAM	Forward	GTT CAG GAC TGG CCC TGC TAA CA
	Chr 5/212-224 bp	Reverse	CCT CCA GCC CAC TTT CTC TTC TC
ETH225	NED	Forward	GAT CAC CTT GCC ACT ATT TCC T
	Chr 9/141-159 bp	Reverse	ACA TGA CAG CCA GCT GCT ACT
ETH3	NED	Forward	GAA CCT GCC TCT CCT GCA TTG G
	Chr 19/105-125 bp	Reverse	ACT CTG CCT GTG GCC AAG TAG G
TGLA53	FAM	Forward	GCT TTC AGA AAT AGT TTG CAT TCA
	Chr 16/152-186 bp	Reverse	ATC TTC ACA TGA TAT TAC AGC AGA
BM1818	HEX	Forward	AGC TGG GAA TAT AAC CAA AGG
	Chr 23/253-272 bp	Reverse	AGT GCT TTC AAG GTC CAT GC
***Additional primer sequences (NCBI)***			
CSRM60	HEX	Forward	AAG ATG TGA TCC AAG AGA GAG GCA
	Chr 10/96-116 bp	Reverse	AGG ACC AGA TCG TGA AAG GCA TAG

Legend: ISAG: International Society for Animal Genetics; NCBI: National Center for Biotechnology Information.

#### Global scoring of DNA concentration, quality of the agarose gel electrophoresis and multiplex-PCR, micro-satellite typing and cattle parentage determination

A global score going from a minimum of 0 and a maximum of 9, was obtained in order to summarize the results of the concentration of DNA, quality of the agarose gel electrophoresis and multiplex-PCR, micro-satellite typing and parentage determination of cattle. For this, the codification was translated for each parameter (0 for Ø [to be excluded], 1 for + [critical]; 2 for ++ [good] and 3 for +++ [excellent]). The global score was defined as the sum of scores for the three above mentioned parameters.

### Statistical analysis

A two-factor ANOVA with repeated measures on one factor was used to compare the kinetic of the optical densities obtained using the E^rns^–based Ag ELISA with dilutions of five positive ear notch samples (BVDV positive PI group of calves) [15]. Four different protocols at three different time periods were used. The validity conditions (homogeneity of variances and covariance matrixes) were preliminary tested [16]. Pairwise comparison between optical densities (OD) obtained in the E^rns^-based Ag ELISA using protocol 2, 3, 4, 5 or 6 and protocol 1 as reference was performed using a two-sample Wilcoxon rank-sum (Mann-Whitney) test and assuming unequal variance and data not distributed as a normal distribution [17]. The same test was used for pairwise comparison of the global score between P4 and P3, P4 and P6, P5 and P3, and P5 and P6, respectively. For all tests, *P*-value < 0.05 was considered significant.

### Ethical approval

Ear notches were sampled at birth by farmers in the context of the mandatory identification of newborn calves and the BVDV eradication program. Complete ear samples were sampled on dead animals or animals euthanized for reasons that were independent from the current study. Both described conditions did not require the approval of an animal ethical committee. Nonetheless, the privacy rights of participants were fully protected and all data were anonymized.

## Results

### Determination of analytical sensitivity of the E^rns^–based Ag ELISA applied on five BVDV positive PI samples prepared with protocols P1, P2, P3 and P4, at day 1, day 3 and day 14

Minimal and median positive dilutions providing a positive result at the ELISA test and obtained with five samples (BVDV positive PI group) are presented in **Table 3**for each protocol and day of testing. Based on the expert opinion of the National Reference Laboratory, the analytical sensitivity of the ELISA performed on BVDV positive samples must be equal or superior to dilution 1:64. The obtained results were at least 2 times this recommended minimal dilution.

**Table 3:** Minimal and median positive dilution levels obtained of five reference samples for each protocol and day of testing.

Protocol	Minimal positive dilution			Median positive dilution		
	Day 1	Day 3	Day 14	Day 1	Day 3	Day 14
P1	512	1024	2048	1024	1024	2048
P2	1024	2048	2048	2048	2048	2048
P3	256	512	1024	512	1024	2048
P4	128	512	1024	512	1024	2048

Legend: The protocols P1 to P4 are described in Table 1.

**Fig 2:**
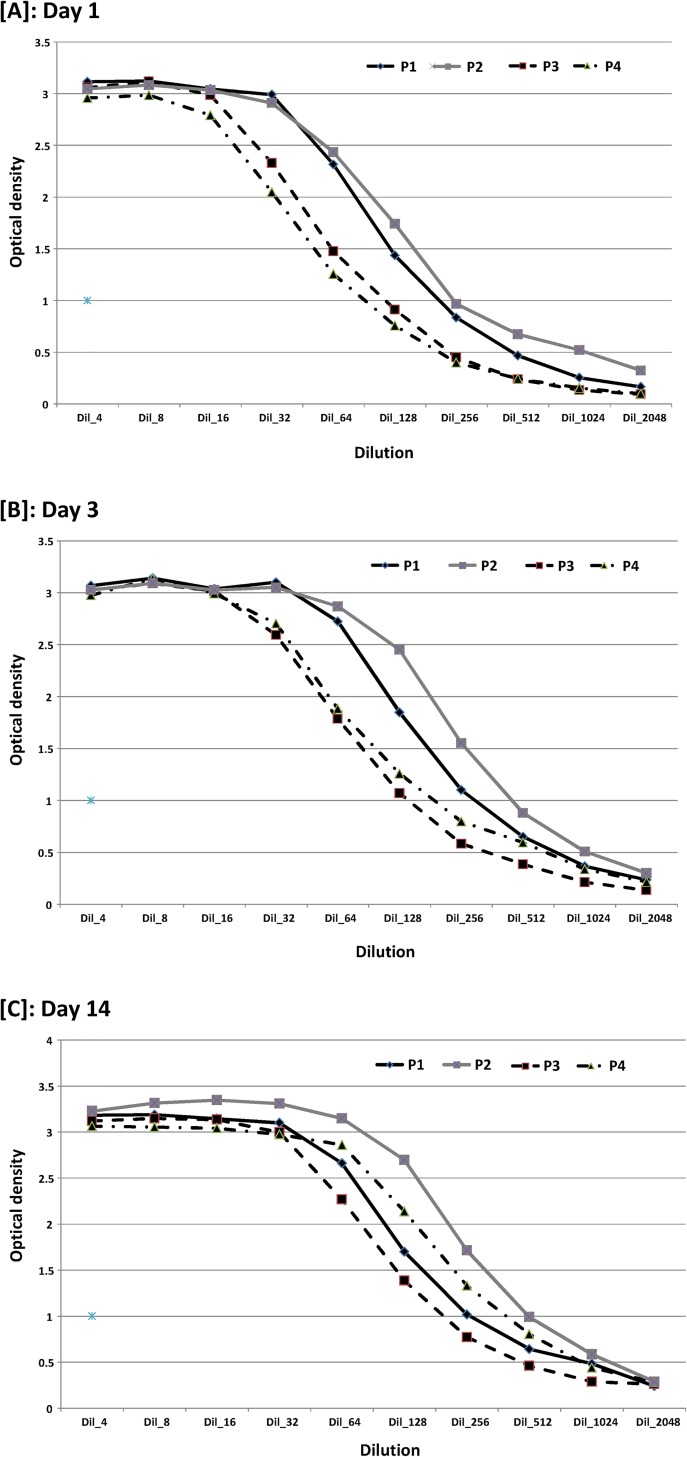
Kinetic of median optical density obtained with the Erns–based Ag ELISA using dilution of five reference samples. **Four different protocols were used at each of the three different time periods (day 1 in A, day 3 in B and day 14 in C).** Legend: P1, P2, P3, P4: protocols applied to the samples; No significant difference between groups was found at day 1 and 14 (two-factor ANOVA with repeated measures on one factor; P-value > 0.05); significant difference between groups (two-factor ANOVA with repeated measures on one factor, P-value < 0.05) was found at day 3.

The trend of the median OD values obtained with the E^rns^–based Ag ELISA is presented in function of the several dilutions of the 5 reference samples and according to the applied protocol (P1, P2, P3 and P4) (**Fig 2**). No statistical difference between the protocols was observed at day 1 and day 14. However, at day 3 the median OD values recorded using P3 and P4 were significantly lower than those recorded using P1 and P2 (two-factor ANOVA with repeated measures on one factor; *P*-value < 0.05). Nevertheless, this difference related to sample preparation method had no influence on the level of detectability and analytical sensitivity of the diagnostic test.

### Determination of the diagnostic specificity of the E^rns^–based Ag ELISA applied on 68 negative samples prepared with protocol P3 and P4 on day 7

A 100% DSp (95% confidence interval [CI]: 95.69–100) was obtained with all tested protocols. The statistics applied to the OD values obtained with the E^rns^-based Ag ELISA are presented in **Table 4**(raw data are depicted in **S1 Table**). These statistics allowed to demonstrate the superiority of P3 and P4 protocols (at day 7) compared to P1 protocol (at day 1) with lower OD values in the E^rns^-based Ag ELISA, regardless of the diluent used (Wilcoxon rank-sum test; *P*-value <0.0001). However, no significant difference (Wilcoxon rank-sum test, *P*-value = 0.13) could be observed between P3 and P4 protocols. OD values recorded with all BVDV negative samples were lower than the negative threshold (OD = 0.200), regardless of the applied protocol.

**Table 4:** Statistics applied to optical densities obtained with the E^rns^–based Ag ELISA on 68 field negative samples, according to the protocol (P) and day (D) used.

	Optical Densities (OD)		
ID	P1, D1	P3, D7	P4, D7
Minimum	0.000	0.000	0.000
Maximum	0.191	0.086	0.155
Average	0.054	0.006	0.022
S.D.	0.036	0.012	0.038
Median	0.051	0.000	0.000

Legend: ID: identification of samples; SD: standard deviation; P1, P3 and P4: protocols (see Table 1); D1 and D7: days after preparation of the sample.

### Evaluation of the diagnostic sensitivity of the E^rns^–based Ag ELISA applied on 30 BVDV ELISA positive samples prepared with protocol P3, P4, P5 and P6 on day 3, 7 and 28

**Fig 3:**
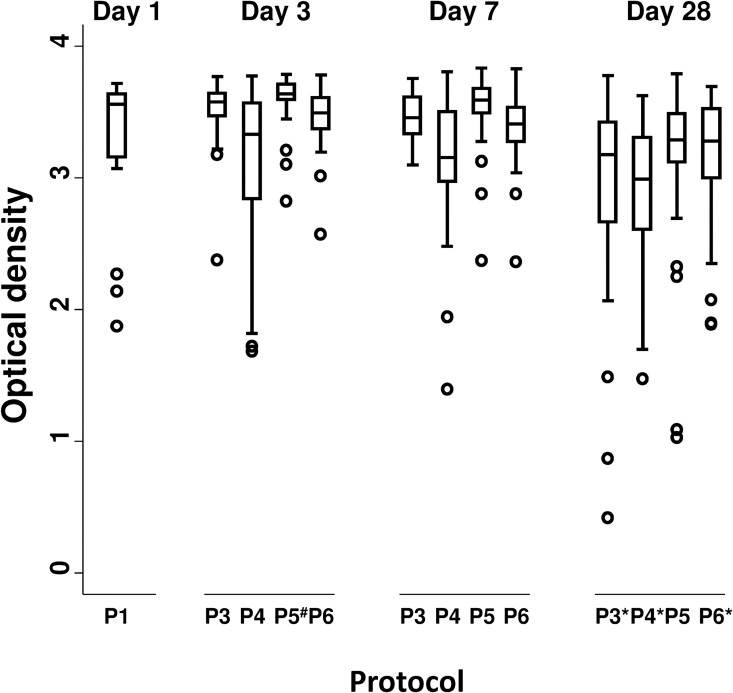
Boxplots of the optical density values obtained with the Erns-based Ag ELISA for different protocols at day 1 (reference), 3, 7 and 28. Legend: P1, P3, P4, P5 and P6: protocols applied to the samples. P1 at day 1 is the reference protocol. ^#^ Optical density (protocol P5 at day 3) is significantly higher than the reference protocol. * Optical density (protocols P3, P4 and P6 at day 28) is significantly lower than the reference protocol.

Whatever the protocol or the day of testing the DSe reached 100% (95% CI: 90.50–100). Indeed, each of the 30 BVDV non IP positive samples (BVDV ELISA positive group) gave a qualitative positive result in the E^rns^–based Ag ELISA (**Fig 3**; raw data are depicted in **S2 Table**). Indeed, the lowest OD value recorded in the E^rns^-based Ag ELISA was in 0.417, largely above the positive threshold (0.300). However, some significant differences in OD were observed between protocols. Protocols P3, P4 and P6 applied at day 28 as well as the P4 protocol applied at day 7 were less efficient than the reference protocol P1 in terms of OD intensity (Wilcoxon rank-sum test; *P*-value < 0.007). On the contrary, OD measured with P5 protocol applied at day 3 were statistically significantly higher than OD obtained with the reference P1 protocol (Wilcoxon rank-sum test; *P*-value = 0.003).

### Assessment of time effect on results

**Fig 4:**
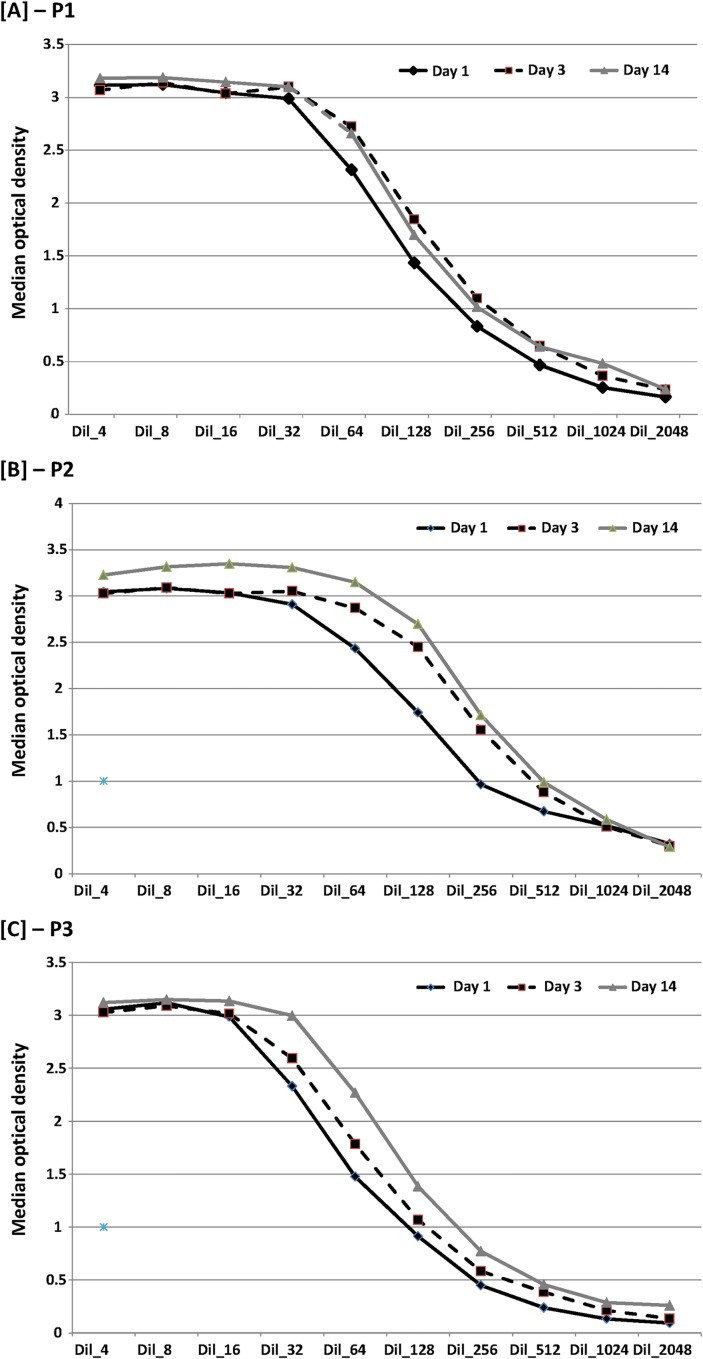
Kinetic of median optical density obtained with the Erns–based Ag ELISA in function of dilution of five positive reference samples at three different time periods for each of the four different protocols used. Legend: P1, P2, P3, P4: protocols applied to the samples; P1 and P3: no significant difference in function of time (two-factor ANOVA with repeated measures on one factor; P-value > 0.05); P2 and P4: significant difference in function of time (two-factor ANOVA with repeated measures on one factor; P-value < 0.05).

The results are presented in **Fig 4**. Fig 4A, 4B, 4C and 4D show the trend of the median OD in the E^rns^–based Ag ELISA in function of the dilutions of the of 5 positive samples (BVDV positive PI group) tested with one protocol (P3, P4, P5 or P6) at different days (D1, D3 and D14). No time effect was observed except for P2 and P4 protocols (two-factor ANOVA with repeated measures on one factor; *P*-value < 0.05).

### Photometric assessment of concentration and purity of DNA

All samples containing adequate DNA concentration showed a clean spectrum of DNA with a maximum absorption at 260 nm and sparse impurity induced by protein, solvent, and salt. **Table 5**gives an overview of the DNA concentration expressed as ng DNA/μl obtained with the samples tested with protocol P3, P4, P5 and P6 (raw data are depicted in **S3 Table**). Four different DNA categories were described (**S3 Table** and summary **Table 6**) and marked as:

- Ø, inadequate DNA concentration (<10ng/μl), failing in most gene-diagnostic examinations,
- +, adequate DNA concentration (10–20 ng/μl), potentially succeeding in simple PCR diagnostic,
- ++, sufficient DNA concentration (20–50 ng/μl), suitable for mostly PCR techniques,
- +++, fulfil the requirements of chip based single nucleotide polymorphism (SNP) genotyping.

**Table 5:** Statistics of photometric determination of DNA concentration.

	DNA ng/μl			
ID	P3	P4*	P5	P6*
Min	0.00	62.27	0.02	5.49
Max	152.31	416.88	118.61	336.77
Average	28.15	259.74	28.57	127.75
S.D.	34.10	90.43	31.21	76.55
Median	18.08	279.26	15.73	115.04

Legend: P3, P4, P5 and P6: protocols applied to the samples; ID: identification of sample; S.D.: standard deviation.

* DNA concentration significantly higher in protocols P4 and P6 in comparison with protocols P3 and P5 (*P*-value < 0.0001).

**Table 6:** DNA concentration, agarose gel electrophoresis and multiplex PCR, micro-satellite typing and cattle parentage determination.

ID	P3				P4**				P5				P6*			
	G. Sr.	conc.	gel	PCR	G. Sr.	conc.	Gel	PCR	G. Sr.	conc.	gel	PCR	G. Sr.	conc.	gel	PCR
1	2	Ø	++	Ø	9	+++	+++	+++	7	++	++	+++	4	Ø	++	++
2	6	+++	Ø	+++	9	+++	+++	+++	7	++	++	+++	6	+++	Ø	+++
3	5	Ø	++	+++	9	+++	+++	+++	6	+	++	+++	7	+++	+	+++
4	3	Ø	Ø	+++	9	+++	+++	+++	0	Ø	Ø	Ø	3	Ø	Ø	+++
5	2	++	Ø	Ø	8	+++	++	+++	8	+++	++	+++	9	+++	+++	+++
6	5	++	Ø	+++	9	+++	+++	+++	4	Ø	+	+++	9	+++	+++	+++
7	2	Ø	Ø	++	9	+++	+++	+++	0	Ø	Ø	Ø	9	+++	+++	+++
8	3	++	Ø	+	9	+++	+++	+++	3	+	Ø	++	9	+++	+++	+++
9	2	++	Ø	Ø	9	+++	+++	+++	1	+	Ø	Ø	9	+++	+++	+++
10	3	+++	Ø	Ø	9	+++	+++	+++	1	+	Ø	Ø	9	+++	+++	+++
11	4	++	++	Ø	9	+++	+++	+++	4	++	++	Ø	9	+++	+++	+++
12	3	Ø	+++	Ø	9	+++	+++	+++	4	+	++	+	9	+++	+++	+++
13	6	+	++	+++	9	+++	+++	+++	6	++	+	+++	9	+++	+++	+++
14	2	Ø	++	Ø	9	+++	+++	+++	2	Ø	++	Ø	9	+++	+++	+++
15	4	Ø	++	++	9	+++	+++	+++	1	+	Ø	Ø	9	+++	+++	+++
16	7	+++	+	+++	9	+++	+++	+++	7	+++	+	+++	9	+++	+++	+++
17	3	Ø	++	+	9	+++	+++	+++	2	Ø	++	Ø	9	+++	+++	+++
18	8	+++	++	+++	9	+++	+++	+++	6	++	+	+++	9	+++	+++	+++
19	2	Ø	++	Ø	9	+++	+++	+++	5	Ø	++	+++	9	+++	+++	+++
20	5	++	Ø	+++	9	+++	+++	+++	7	+++	+	+++	8	++	+++	+++
21	2	Ø	++	Ø	9	+++	+++	+++	6	++	+	+++	9	+++	+++	+++
22	5	++	Ø	+++	9	+++	+++	+++	1	Ø	+	Ø	9	+++	+++	+++
23	7	+++	+	+++	9	+++	+++	+++	0	Ø	Ø	Ø	9	+++	+++	+++
24	6	++	+	+++	9	+++	+++	+++	7	+++	+	+++	9	+++	+++	+++
25	2	Ø	++	Ø	9	+++	+++	+++	2	Ø	++	Ø	9	+++	+++	+++
26	2	Ø	++	Ø	9	+++	+++	+++	7	+++	+	+++	8	++	+++	+++
27	5	++	Ø	+++	9	+++	+++	+++	5	++	Ø	+++	9	+++	+++	+++
28	3	Ø	++	+	9	+++	+++	+++	4	+	Ø	+++	9	+++	+++	+++
29	4	++	++	Ø	9	+++	+++	+++	2	Ø	++	Ø	9	+++	+++	+++
30	2	Ø	++	Ø	9	+++	+++	+++	0	Ø	Ø	Ø	9	+++	+++	+++
Min	2				8				0				3			
Median	3				9				4				9			
Max	8				9				8				9			
Average	3.83				8.97				3.83				8.40			
S.D.	1.82				0.18				2.65				1.50			

Legend: P: protocol considered; Min: Minimum; Max: Maximum; S.D.: Standard deviation; conc.: DNA concentration; gel: agarose gel electrophoresis; PCR: Multiplex-PCR, micro-satellite typing and cattle parentage determination; codification: Ø (excluded [in dark red]), + (critical [in pink]), ++ (good [in light green]) and +++ (excellent [in dark green]); G.Sc.: the global score is the result of the sum of scores of DNA concentration, agarose gel electrophoresis and micro-satellite typing and cattle parentage determination (after transformation of Ø, +, ++ and +++ as 0, 1, 2 and 3, respectively)

*: significantly higher than protocols P3 and P5 (*P*-value < 0.0001)

**: significantly higher than protocols P3, P5 (*P*-value < 0.0001) and P6 (*P*-value = 0.04).

In average the protocols P4 and P6 (using Allflex buffer) allowed to obtain 9 and 4 times more DNA than the protocols P3 and P5 (using IDEXX buffer). This difference was significant (two sample Wilcoxon rank sum; *P*-value < 0.0001). Despite the absence of significant difference between protocols P3 and P5 (two sample Wilcoxon rank sum; *P*-value = 0.70), the quantity of DNA was significantly higher in protocol P4 in comparison with the protocol P6 (two sample Wilcoxon rank sum; *P*-value < 0.0001).

### Genomic DNA using agarose gel electrophoresis

In order to evaluate degradation of DNA according to the used protocol, one aliquot of each DNA sample was examined by electrophoresis. **S1 Fig** shows the agarose gel pictures of all samples tested with the different four protocols P3, P4, P5 and P6.

While all samples of protocol P4 (30 of 30) and most of P6 (27 of 30) showed a conspicuous high-molecular DNA band, only few of the samples of protocol P3 (16 of 30) and even less of P5 (11 of 30) did. Most of P5 samples showed a low-molecular DNA band, corresponding to a high portion of fragmented DNA.

Four categories of quality of agarose gel electrophoresis (see summary **Table 6**): Ø, for samples with no detectable high-molecular band; +, for samples with a bright smear band or clear DNA degradation; ++, for samples with a small high-molecular band and +++, for samples with complete intact DNA shown represented by a dense high-molecular band.

### Multiplex-PCR, micro-satellite typing and cattle parentage determination

The routine micro-satellite typing amplification, as internationally applied for cattle parentage determination, was outstanding in all samples of protocols P4 and P6. **Table 6**represents the summary of results. For the samples of protocols P4 and P6, no inhibition of gene-diagnostic methods by contaminating agents was observed. Four categories were defined in function of the result of the multiplex PCR: +++, when all markers (13 of 13) were amplified; ++, if 1 up to 3 markers of 13 did not amplify in a sample; +, if 4 up to 12 markers of 13 did not amplify in a sample and Ø, when the amplification failed completely, i.e. a lot of samples of protocols P3 (13 of 30) and P5 (13/30).

### Global scoring of DNA concentration, quality of the agarose gel electrophoresis and multiplex-PCR, micro-satellite typing and parentage determination of cattle

The global score was significantly higher using protocols P4 and P6 (Allflex buffer) in comparison with protocols P3 and P5 (IDEXX buffer) (two-sample Wilcoxon rank-sum; P<0.0001). Despite the absence of significant difference between protocols P3 and P5 (two-sample Wilcoxon rank-sum; *P*-value = 0.95), the global score was significantly higher using P4 in comparison with P6 (two-sample Wilcoxon rank-sum; *P*-value = 0.04).

## Discussion

The Belgian official BVD eradication programme is based on the detection by ELISA test of the BVDV E^rns^ protein on ear notches of newborn calves at birth. The diagnostic method is imposed by the legislation using a specific sample diluent (IDEXX buffer, ear notch tissue soaking buffer of the IDEXX indirect antigen capture BVDV Ag/Serum Plus Test). However this diluent is not suitable for storing and genetic characterization of the animal DNA.

The first objective of this study was to test another diluent, the Allflex DNA Buffer D (Allflex buffer), recognized for effective conservation and subsequent genetic analysis of animal DNA (data available on request to Allflex). Secondly, this study wanted to verify the impact of dilutions on diagnostic characteristics of the E^rns^–based Ag ELISA test.

In general, detectability, analytical sensitivity, DSe and DSp were not affected by the use of Allflex buffer in terms of BVDV qualitative diagnostic interpretation. However, some significant quantitative differences were found depending on the protocol, the analysis period or the buffer used. These findings should be further confirmed in a large scale study. This buffer, by protection of DNA contained within the eluent of biopsy, improves laboratory logistics by using a pre-filled tube containing the eluent to enclose the needle component of the TST. In this way, fastidious and time consuming steps associated with the extraction of the biopsy out of the sampling system can be avoided.

Furthermore, when the contact time between the sample and the buffer was extended, an improvement of the elution sample was expected.

Based on expert opinion produced by the Belgian National Reference Laboratory, the analytical sensitivity of the ELISA performed on BVDV positive samples must be equal or superior to dilution 1:64. All tested samples (obtained from the BVDV positive PI group) showed positive results until dilution 1:128. Thus, all protocols had acceptable analytical sensitivity, although a significantly lower OD was measured with protocols P3 and P4. In P3 and P4, at the time of the ear tag application, the needle was closed in a tube pre-filled with IDEXX or Allflex buffer. These differences were probably more related to the protocol itself and not to the liquid used since these differences were observed with both liquids.

The DSp was 100% for all protocols (95% CI: 95.69–100) and seemed not affected by either the protocol neither the diluent used. However, P3 and P4 protocols had significantly lower OD in the E^rns^–based Ag ELISA in comparison with protocol P1 at day 1 as reference, but no significant difference was observed between the two buffers. Despite a possible reconsideration of the cut-off of the E^rns^–based Ag ELISA based on the notice of the producer, this observation can be of importance in case of the use of the protocol P3 or P4 in a more large population (i.e. a more important specificity is expected).

Finally, all the BVDV positive samples (from both BVDV positive PI and BVDV ELISA positive groups) remained positive whatever the protocol or the delay between the preparation of the sample and the test (expressed in days). This result confirmed the good DSe of the test although the sampling was performed on dead or euthanized animals. Although effective on field positive samples, the ELISA test carried out at day 28 with the protocols P3, P4 and P6 showed significantly lower OD than the reference protocol P1. This result may be surprising, since the contact time between the sample and the buffer was extended and was expected to improve elution sample. However, the values of all ODs for alternative protocols were largely above the cut-off recommended by the producer and the impact on DSe could be low.

The influence of the Allflex buffer on the results of negative samples using P3 and P4 protocols at day 7 did not affect the accuracy of the qualitative result interpretation.

In summary, replacement of IDEXX buffer by Allflex buffer in the ear notch preparation procedure did not affect quality or accuracy of the diagnostic test used in the current official Belgian BVDV eradication programme. Moreover, additional tests and animal genetic characterization according to the different protocols on day 28 allowed to demonstrate the higher performance of Allflex buffer in comparison to the IDEXX buffer. Samples analysed after the use of protocols P3, P4, P5 and P6 showed a wide range of DNA concentration. This outcome may be attributable to variation in the DNA concentration inherent to the tissue samples or/and to handling variation in individual laboratory staff despite that all lab routines and examinations being carried out according to standardized procedures. Samples prepared protocols with P4 and P6 (Allflex buffer) contained an average of 4 to 9 times more DNA than those prepared with the P3 and P5 protocols (IDEXX buffer) and this significant difference was related to the liquid rather than protocol.

In addition, DNA quality, PCR amplification and micro-satellites typing showed a striking superiority of Allflex buffer to the IDEXX buffer. Again, the overall score was widely and significantly higher for the P4 and P6 protocols (Allflex buffer) in comparison with protocols P3 and P5 (IDEXX buffer). These results confirm therefore the expected qualities of Allflex buffer to preserve and conserve intact animal DNA in a sample, even from a dead or euthanized animal.

Allflex buffer opens to the perspective of sample traceability by genome conservation. DNA is a permanent biometric marker successfully used for pedigree verification, parental identification and breeding programmes [18], retrospective audits and meat tracing [19]. It could be also used in the Belgian cattle population to estimate the proportion of erroneous identification of animal sampled during the BVD eradication programme and to determine the breed of cattle from which tissue samples are obtained [20]. The DNA could be extracted and analysed on a SNP microarray chip technology [21] and results could be transmitted to a database. As SNPs can be tested in great numbers it could give the opportunity to detect patterns or genetic signatures throughout the genome associated with particular traits [22]. SNPs are the marker of choice for the cattle industry because animal with valid genotypes could be evaluated and genomic breeding values could be generated. In UK, cattle testing positive for bovine tuberculosis are immediately DNA tagged, and the DNA sample is retained by Animal Health service and cross-checks against animals sent for slaughter are made (http://www.defra.gov.uk/news/2011/03/31/cattle-bovine-tb/).

In summary, the method proposed in this study allows saving time and funds as only one sample could be used for both BVDV evaluation and DNA extraction permitting to ensure traceability.

Currently, a wide work of DNA storage is done in Ireland but based on two separate ear samples per animal [23–24], implying the use of the double tags and a double laboratory manipulation. Parentage verification, genomic selection, testing of known major genes (fertility haplotypes (HH1, HH2, HH3, JH1), A1/A2 beta casein, MSTN, DGAT1), lethal recessives (BLAS, CVM, DUMPS, brachyspina) or congenital disorders (e.g. curly/fawn calf, spiderleg, tibial hemimelia, hairlessness (hypotrichosis), scurs, mulefoot, red recessive coat colour) are targeted by the Irish Ministry. Moreover, the data base can be annually updated [21].

## Supporting Information

S1 Fig Electropherograms of DNA contents of random 12 positive and 18 negative field samples. Legend: P3, P4, P5 and P6: protocols applied to the samples; Leer: empty; Identification of samples is on the right of each electropherogram.
(TIF)

S1 Table Distribution of optical density obtained with the E^rns^–based Ag ELISA performed on 68 field negative samples according to the protocol (P) and the day (D). Legend: ID: identification of samples; SD: standard deviation; P1, P3 and P4: protocols applied to the samples; D1 and D7: days after preparation of the sample.
(DOCX)

S2 Table Distribution of optical density obtained with the Erns–based Ag ELISA of 30 positive samples from the BVDV ELISA positive group of calves according to the protocol and the day after preparation. Legend: ID: identification of samples; SD, standard deviation; P1, P3, P4, P5 and P6: protocols applied to the samples; D1, D3, D7, D28: days after preparation of the sample; Av.: average; Med.: median; -: not determined.
(DOCX)

S3 Table Photometric determination of DNA concentration and purity. Legend: P3, P4, P5 and P6: protocols applied to the samples; ID sample: identification of sample; -: not determined; SD: standard deviation; Background colors are used to identify the samples based on their DNA concentration and purity (dark red with inadequate DNA concentration (<10ng/μl) doomed to fail most gene-diagnostic examinations; light red with adequate DNA concentration (10–20 ng/μl) potentially to succeed in simple PCR diagnostics; light green with sufficient DNA concentration (20–50 ng/μl) for complex PCR methods; dark-green when concentration and purity fulfill the requirements of chip based SNP typing (Illumina).
(DOCX)
