# Supplementary material for: First Results in the Use of Bovine Ear Notch Tag for Bovine Viral Diarrhoea Virus Detection and Genetic Analysis
Source: PLoS One. 2016 Oct 20;11(10):e0164451. doi: 10.1371/journal.pone.0164451 (PMC5072587; doi:10.1371/journal.pone.0164451)
Supplement: S1 Table — Legend: ID: identification of samples; SD: standard deviation; P1, P3 and P4: protocols applied to the samples; D1 and D7: days after preparation of the sample. (DOCX) [file pone.0164451.s002.docx]

**S1 Table. Distribution of optical density corrected in the E^rns^–based Ag ELISA of 68 samples coming from the negative control group according to the protocol (P) and day (D) used**

| ID | P1, D1 | P3, D7 | P4, D7 |
| --- | --- | --- | --- |
| 1 | 0.010 | 0.014 | 0.094 |
| 2 | 0.047 | 0.011 | 0.054 |
| 3 | 0.092 | 0.016 | 0.065 |
| 4 | 0.037 | 0.020 | 0.119 |
| 5 | 0.034 | 0.025 | 0.052 |
| 6 | 0.011 | 0.014 | 0.055 |
| 7 | 0.011 | 0.017 | 0.111 |
| 8 | 0.024 | 0.010 | 0.063 |
| 9 | 0.005 | 0.011 | 0.090 |
| 10 | 0.010 | 0.012 | 0.002 |
| 11 | 0.083 | 0.020 | 0.032 |
| 12 | 0.034 | 0.026 | 0.061 |
| 13 | 0.191 | 0.016 | 0.051 |
| 14 | 0.029 | 0.011 | 0.035 |
| 15 | 0.058 | 0.018 | 0.038 |
| 16 | 0.007 | 0.000 | 0.155 |
| 17 | 0.024 | 0.006 | 0.153 |
| 18 | 0.022 | 0.020 | 0.049 |
| 19 | 0.000 | 0.010 | 0.036 |
| 20 | 0.077 | 0.086 | 0.079 |
| 21 | 0.030 | 0.009 | 0.010 |
| 22 | 0.053 | 0.002 | 0.000 |
| 23 | 0.064 | 0.005 | 0.000 |
| 24 | 0.061 | 0.002 | 0.000 |
| 25 | 0.051 | 0.000 | 0.000 |
| 26 | 0.080 | 0.000 | 0.000 |
| 27 | 0.101 | 0.010 | 0.000 |
| 28 | 0.118 | 0.001 | 0.004 |
| 29 | 0.035 | 0.000 | 0.012 |
| 30 | 0.170 | 0.000 | 0.017 |
| 31 | 0.077 | 0.002 | 0.030 |
| 32 | 0.079 | 0.000 | 0.005 |
| 33 | 0.072 | 0.000 | 0.003 |
| 34 | 0.076 | 0.000 | 0.000 |
| 35 | 0.109 | 0.000 | 0.000 |
| 36 | 0.013 | 0.000 | 0.005 |
| 37 | 0.062 | 0.000 | 0.000 |
| 38 | 0.085 | 0.000 | 0.000 |
| 39 | 0.059 | 0.000 | 0.000 |
| 40 | 0.051 | 0.000 | 0.000 |
| 41 | 0.101 | 0.000 | 0.000 |
| 42 | 0.070 | 0.000 | 0.000 |
| 43 | 0.010 | 0.000 | 0.000 |
| 44 | 0.049 | 0.000 | 0.000 |
| 45 | 0.068 | 0.000 | 0.027 |
| 46 | 0.066 | 0.000 | 0.000 |
| 47 | 0.046 | 0.000 | 0.000 |
| 48 | 0.065 | 0.000 | 0.000 |
| 49 | 0.093 | 0.000 | 0.000 |
| 50 | 0.094 | 0.000 | 0.000 |
| 51 | 0.033 | 0.000 | 0.000 |
| 52 | 0.059 | 0.000 | 0.000 |
| 53 | 0.046 | 0.000 | 0.000 |
| 54 | 0.046 | 0.000 | 0.000 |
| 55 | 0.049 | 0.000 | 0.000 |
| 56 | 0.066 | 0.000 | 0.000 |
| 57 | 0.050 | 0.000 | 0.000 |
| 58 | 0.064 | 0.000 | 0.000 |
| 59 | 0.032 | 0.002 | 0.000 |
| 60 | 0.046 | 0.004 | 0.013 |
| 61 | 0.038 | 0.000 | 0.000 |
| 62 | 0.019 | 0.000 | 0.000 |
| 63 | 0.038 | 0.000 | 0.000 |
| 64 | 0.038 | 0.000 | 0.000 |
| 65 | 0.052 | 0.000 | 0.000 |
| 66 | 0.073 | 0.000 | 0.000 |
| 67 | 0.024 | 0.000 | 0.000 |
| 68 | 0.014 | 0.000 | 0.000 |

Legend: ID: identification of samples; SD: standard deviation; P1, P3 and P4: protocols applied to the samples; D1 and D7: days after preparation of the sample.
