# Supplementary material for: First Results in the Use of Bovine Ear Notch Tag for Bovine Viral Diarrhoea Virus Detection and Genetic Analysis
Source: PLoS One. 2016 Oct 20;11(10):e0164451. doi: 10.1371/journal.pone.0164451 (PMC5072587; doi:10.1371/journal.pone.0164451)
Supplement: S2 Table — Legend: ID: identification of samples; SD, standard deviation; P1, P3, P4, P5 and P6: protocols applied to the samples; D1, D3, D7, D28: days after preparation of the sample; Av.: average; Med.: median; -: not determined. (DOCX) [file pone.0164451.s003.docx]

**S2 Table. Distribution of optical density corrected in the Erns–based Ag ELISA of 30 samples coming from the BVDV ELISA positive group according to the protocol and test day used**

| ID | P1 | P3 | | | P4 | | | P5 | | | P6 | | |
| --- | --- | --- | --- | --- | --- | --- | --- | --- | --- | --- | --- | --- | --- |
|  | D1 | D3 | D7 | D28 | D3 | D7 | D28 | D3 | D7 | D28 | D3 | D7 | D28 |
| 1 | 3.609 | 3.566 | 3.460 | - | 3.481 | 3.498 | 3.523 | 3.546 | 3.691 | 3.288 | 3.582 | 3.536 | 3.564 |
| 2 | 3.638 | 3.570 | 3.552 | 0.417 | 3.029 | 3.154 | 2.918 | 3.616 | 3.703 | 3.283 | 3.467 | 3.540 | 3.400 |
| 3 | 3.393 | 3.221 | 3.236 | 2.691 | 2.289 | 1.942 | 1.764 | 3.102 | 3.125 | 1.086 | 2.570 | 2.361 | 1.887 |
| 4 | 3.557 | 3.363 | 3.388 | 0.869 | 3.568 | 3.560 | 3.544 | 3.211 | 3.273 | 1.026 | 3.579 | 3.571 | 3.540 |
| 5 | 3.527 | 3.559 | 3.295 | 3.243 | 3.076 | 2.975 | 3.113 | 3.636 | 3.489 | 3.434 | 3.523 | 3.471 | 3.396 |
| 6 | 3.104 | 3.643 | 3.360 | 3.518 | 3.568 | 3.022 | 3.309 | 2.825 | 2.374 | 2.696 | 3.407 | 2.879 | 3.150 |
| 7 | 3.070 | 3.667 | 3.345 | 3.426 | 1.818 | 2.543 | 2.519 | 3.710 | 3.374 | 3.591 | 3.599 | 3.442 | 3.509 |
| 8 | 1.874 | 3.471 | 3.457 | 2.335 | 3.368 | 3.405 | 2.200 | 3.549 | 3.683 | 2.691 | 3.282 | 3.363 | 2.350 |
| 9 | 3.665 | 3.500 | 3.480 | 2.666 | 3.384 | 3.390 | 3.124 | 3.630 | 3.547 | 3.203 | 3.424 | 3.321 | 3.142 |
| 10 | 3.717 | 3.692 | 3.648 | 3.327 | 3.354 | 3.100 | 2.521 | 3.719 | 3.680 | 3.274 | 3.401 | 3.277 | 2.933 |
| 11 | 3.610 | 3.562 | 3.286 | 1.490 | 2.983 | 3.133 | 2.920 | 3.744 | 3.607 | 3.365 | 3.399 | 3.350 | 3.093 |
| 12 | 3.372 | 3.378 | 3.410 | 2.890 | 1.684 | 1.395 | 1.473 | 3.448 | 3.506 | 2.827 | 3.366 | 3.292 | 2.691 |
| 13 | 3.668 | 3.178 | 3.170 | 2.710 | 2.381 | 2.905 | 2.833 | 3.562 | 3.546 | 3.487 | 3.015 | 3.126 | 2.998 |
| 14 | 3.657 | 3.643 | 3.686 | 3.379 | 3.717 | 3.607 | 3.444 | 3.768 | 3.679 | 3.423 | 3.750 | 3.700 | 3.400 |
| 15 | 3.497 | 3.613 | 3.672 | 3.263 | 2.514 | 3.053 | 2.996 | 3.737 | 3.615 | 3.397 | 3.605 | 3.706 | 3.355 |
| 16 | 2.138 | 2.377 | 3.273 | 2.589 | 2.219 | 2.787 | 2.868 | 3.612 | 3.591 | 2.325 | 3.195 | 3.229 | 1.898 |
| 17 | 3.565 | 3.389 | 3.337 | 2.759 | 1.719 | 3.058 | 3.048 | 3.675 | 3.536 | 3.405 | 3.249 | 3.33 | 3.203 |
| 18 | 3.488 | 3.466 | 3.413 | 3.282 | 2.953 | 2.974 | 2.934 | 3.611 | 3.596 | 3.257 | 3.373 | 3.386 | 3.235 |
| 19 | 3.202 | 3.581 | 3.456 | 3.177 | 3.306 | 3.150 | 2.987 | 3.676 | 3.492 | 3.120 | 3.280 | 3.097 | 3.278 |
| 20 | 3.100 | 3.582 | 3.523 | 2.979 | 3.392 | 3.225 | 2.608 | - | - | - | 3.390 | 3.429 | 3.032 |
| 21 | 2.271 | 3.542 | 3.722 | 3.184 | 3.168 | 3.585 | 3.089 | 3.596 | 3.691 | 3.281 | 3.455 | 3.609 | 2.638 |
| 22 | 3.672 | 3.637 | 3.754 | 3.602 | 3.732 | 3.806 | 3.556 | 3.603 | 3.832 | 3.634 | 3.671 | 3.830 | 3.577 |
| 23 | 3.134 | 3.604 | 3.154 | 2.221 | 3.197 | 2.710 | 1.941 | 3.614 | 3.306 | 3.194 | 3.611 | 3.254 | - |
| 24 | 3.157 | 3.535 | 3.098 | 2.063 | 2.844 | 2.477 | 1.697 | 3.668 | 2.88 | 2.252 | 3.625 | 3.039 | 2.074 |
| 25 | 3.574 | 3.623 | 3.493 | 3.617 | 3.507 | 3.245 | 2.619 | 3.709 | 3.538 | 3.615 | 3.576 | 3.351 | 3.527 |
| 26 | 3.571 | 3.696 | 3.519 | 3.749 | 3.771 | 3.491 | 3.625 | 3.721 | 3.529 | 3.793 | 3.678 | 3.46 | 3.647 |
| 27 | 3.712 | 3.702 | 3.512 | 3.777 | 3.661 | 3.501 | 3.466 | 3.721 | 3.611 | 3.754 | 3.659 | 3.488 | 3.696 |
| 28 | 3.690 | 3.62 | 3.614 | 3.673 | 3.686 | 3.592 | 3.094 | 3.662 | 3.678 | 3.432 | 3.572 | 3.488 | 3.386 |
| 29 | 3.614 | 3.727 | 3.713 | 2.696 | 3.674 | 3.610 | 3.571 | 3.678 | 3.735 | 3.632 | 3.615 | 3.553 | 3.584 |
| 30 | 3.614 | 3.768 | 3.657 | 3.631 | 3.730 | 3.578 | 3.288 | 3.787 | 3.710 | 3.781 | 3.783 | 3.508 | 3.638 |

Legend: ID: identification of samples; SD, standard deviation; P1, P3, P4, P5 and P6: protocols applied to the samples; D1, D3, D7, D28: days after preparation of the sample; Av.: average; Med.: median; -: not determined.
