# Supplementary material for: First Results in the Use of Bovine Ear Notch Tag for Bovine Viral Diarrhoea Virus Detection and Genetic Analysis
Source: PLoS One. 2016 Oct 20;11(10):e0164451. doi: 10.1371/journal.pone.0164451 (PMC5072587; doi:10.1371/journal.pone.0164451)
Supplement: S3 Table — Legend: P3, P4, P5 and P6: protocols applied to the samples; ID sample: identification of sample; -: not determined; SD: standard deviation; Background colors are used to identify the samples based on their DNA concentration and purity (dark red with inadequate DNA concentration (<10ng/μl) doomed to fail most gene-diagnostic examinations; light red with adequate DNA concentration (10–20 ng/μl) potentially to succeed in simple PCR diagnostics; light green with sufficient DNA concentration (20–50 ng/μl) for complex PCR methods; dark-green when concentration and purity fulfill the requirements of chip based SNP typing (Illumina). (DOCX) [file pone.0164451.s004.docx]

**S3 Table. Photometric determination of DNA concentration and purity**

|  | P3 | P4 | P5 | P6 |
| --- | --- | --- | --- | --- |
| ID sample | ng DNA/ul | ng DNA/ul | ng DNA/ul | ng DNA/ul |
| 1 | 0.00 | 222.47 | 23.49 | 5.49 |
| 4 | 89.86 | 310.65 | 36.93 | 116.47 |
| 5 | 4.49 | 176.58 | 14.65 | 87.32 |
| 6 | 0.00 | 416.88 | 1.06 | 6.77 |
| 26 | 22.76 | 345.98 | 56.61 | 195.06 |
| 29 | 38.12 | 276.21 | 3.7 | 108.85 |
| 30 | 3.46 | 262.84 | - | 51.52 |
| 62 | 43.4 | 355.76 | 19.56 | 237.4 |
| 63 | 42.59 | 340.99 | 12.98 | 198.59 |
| 66 | 78.65 | 318.46 | 16.71 | 336.77 |
| 69 | 31.54 | 359.34 | 23.7 | 117.82 |
| 71 | 8.39 | 208.9 | 14.74 | 155.37 |
| 73 | 14.77 | 333.21 | 49.98 | 140.92 |
| 74 | 3.86 | 384.75 | 3.68 | 113.6 |
| 75 | 8.7 | 351.5 | 13.79 | 301.14 |
| 76 | 152.31 | 291.06 | 59.5 | 63.87 |
| 77 | 7.58 | 243 | 0.83 | 195.77 |
| 78 | 65.4 | 160.63 | 29.69 | 134.53 |
| 79 | 1.92 | 336.19 | 6.39 | 90.61 |
| 80 | 40.26 | 160.91 | 92.36 | 28.22 |
| 81 | 3.38 | 144.62 | 43.5 | 171.47 |
| 82 | 21.39 | 210.5 | 4.66 | 83.95 |
| 83 | 56.01 | 309.78 | - | 77.98 |
| 84 | 29.06 | 154.81 | 118.61 | 107.46 |
| 85 | 1.48 | 282.3 | 7.54 | 160.06 |
| 86 | 0.39 | 62.27 | 94.87 | 45 |
| 87 | 42.68 | 135.32 | 35.43 | 127.91 |
| 88 | 0.02 | 198.68 | 14.42 | 152.07 |
| 89 | 26.6 | 133.19 | 0.56 | 109.9 |
| 90 | 5.46 | 304.43 | 0.02 | 110.75 |

Legend: P3, P4, P5 and P6: protocols applied to the samples; ID sample: identification of sample; -: not determined; SD: standard deviation; Dark red marks the samples with inadequate DNA concentration (<10ng/μl) doomed to fail most gene-diagnostic examinations; Light red marks the samples with adequate DNA concentration (10-20 ng/μl) potentially to succeed in simple PCR diagnostics; Light green marked samples (20-50 ng/μl) contain sufficient DNA qualifying for complex PCR methods; Dark-green marked samples fulfill the requirements of chip based SNP typing (Illumina).
